# Resolution of Chronic Migraine Headaches and Improvement in Cervical Spine Kyphosis Following Chiropractic BioPhysics® (CBP®) Treatment: A Case Report With a Seven-Month Follow-Up

**DOI:** 10.7759/cureus.69935

**Published:** 2024-09-22

**Authors:** Paul A Oakley, Jason W Haas, Thomas J Woodham, Miles O Fortner, Deed E Harrison

**Affiliations:** 1 Kinesiology and Health Science, York University, Toronto, CAN; 2 Chiropractic, Innovative Spine and Wellness, Newmarket, CAN; 3 Research, CBP (Chiropractic Biophysics) Non-profit, Inc., Windsor, USA; 4 Chiropractic Biophysics, Western Plains Chiropractic, Gillette, USA; 5 Physical Medicine and Rehabilitation, CBP (Chiropractic Biophysics) Non-profit, Inc., Eagle, USA

**Keywords:** sagittal balance, chiropractic biophysics, spine alignment, cervical lateral radiograph, migraine, cervical lordosis, headache

## Abstract

We present a chronic migraine (CM) patient demonstrating significant improvement in subjective and objective reported outcome measures with deeper cervical lordosis parameters and reduced forward head posture on radiographs. A 29-year-old male suffered from CM reporting significant pain and disability with aural, sensory, and motor disturbances during the migraine headaches. Aura with visual disturbances, abnormal facial and extremity sensation, sporadic motor weakness, and other signs of CM were found in the patient's history since age 10. The patient reported previous physical therapy, manual chiropractic, and over-the-counter medications. Migraine-specific prescriptions without long-term reduction in pain and disability were reported. The pain and suffering had been reported to be worsening, and he sought Chiropractic BioPhysics^®^ (CBP^®^) spine and postural rehabilitation protocols. These protocols were used to increase cervical lordosis, reduce coronal imbalances, increase mobility, and create better posture. These protocols include specific prescriptions based on radiography for postural exercises, postural mirror image^®^ (MI^®^)traction, and specific spinal manipulative therapy (SMT) focused on posture. All outcome measures improved with the resolution of all initial symptoms of CM. There was a 16° improvement in cervical lordosis, a 30% decrease in headache disability, and additional improvements. These improvements were maintained at a seven-month follow-up during which the patient received infrequent maintenance treatments. This successful treatment of a patient with CM with long-term follow-up adds to evidence that CBP^®^ spinal structural rehabilitation may prove effective and serve as a possible tool for clinicians, physicians, and therapists to treat CM.

## Introduction

Chronic migraine (CM) headache is a rare cause of disability affecting up to 1%-2% of the global population and contributes to the global burden of disease (GBD) [1,2]. Migraine causes significant human suffering and dysfunction, reduces productivity, and increases the years lived with disability (YLDs). Although drug therapy is commonly used for the treatment of CM, there are no long-term studies on the cost-benefit analyses of using pharmacologic agents to treat CM [3-5].

Alternative treatments for CM are varied, but most lack strong evidence for efficacy and, particularly, long-term efficacy [6]. A recent case reported that increasing cervical lordosis was effective in resolving migraine, specifically, sporadic hemiplegic migraine [7]. Another study reported the resolution of cervicogenic headaches by increasing the cervical lordosis [8], where the authors suggested that increasing the cervical lordosis via extension traction methods may be a viable treatment for patients suffering from headaches with simultaneous cervical hypolordosis.

This is a case report of a patient with CM who had unsuccessful medication treatment and sought an alternative therapeutic approach with a Chiropractic BioPhysics® (CBP®) rehabilitation facility, which employed cervical extension traction methods as part of a multimodal rehabilitation program as previously reported [7,8]. We also report a seven-month follow-up of all initial evaluation and outcome measures.

## Case presentation

Patient history, and subjective and objective clinical findings

A 29-year-old male suffered from CM for 19 years with an insidious onset at approximately the age of 10. There was no reported history of trauma, injuries, or familial causes known by the patient. The early migraines were severe and debilitating, which caused significant interference with quality of life and school productivity. The CM would always be preceded by an aura that included altered sensations, especially auditorily and visually. The aural, visual, and auditory altered sensation symptoms would sometimes be brief and sometimes would last several hours but were always followed by severe head and face pain. The pain was frequently so severe that his ability to function was significantly hindered and he was forced to be in a dark room and bed for many hours and days over 19 years before the successful treatment reported here. The head pain would also be accompanied by motor dysfunction in his extensor muscles, leading to severe lower extremity cramping and spasms. The patient had been to doctors for the headaches for many years and was frequently taking high doses of over-the-counter (OTC) medications and had previously been prescribed a beta blocker briefly with no benefit. The patient reported prior physical therapy (PT) designed to attempt to strengthen the neck muscles without relief. The “chin tuck” exercise prescribed by the PT as an attempt to correct his posture exacerbated the symptoms. He sought out chiropractors who performed spinal manipulative therapy (SMT) on his neck and reported experiencing only short-term increases in cervical range of motion (ROM) and slight reductions in neck pain (NP). No previous treatments, pharmacological, traditional chiropractic, or PT resulted in a long-term resolution of the CM.

Before seeking care with the CBP® physicians providing the protocol described in this case, the patient reported that the frequency of the CMs was increasing to as many as three days per week with pain and often needed a recovery day following the CM and associated symptoms. The patient reported worsening pain at the occipital muscles bilaterally during migraine, and the cervical spine pain was worsening and radiating into the thoracic spine and musculature. The cramping was worsening and was moving from the lower extremity cephalad into the lower lumbar spine and paraspinal muscles. He was unable to work because of the pain, dysfunction, and disability caused by the CM symptoms.

Radiographic findings

**Figure 1:**
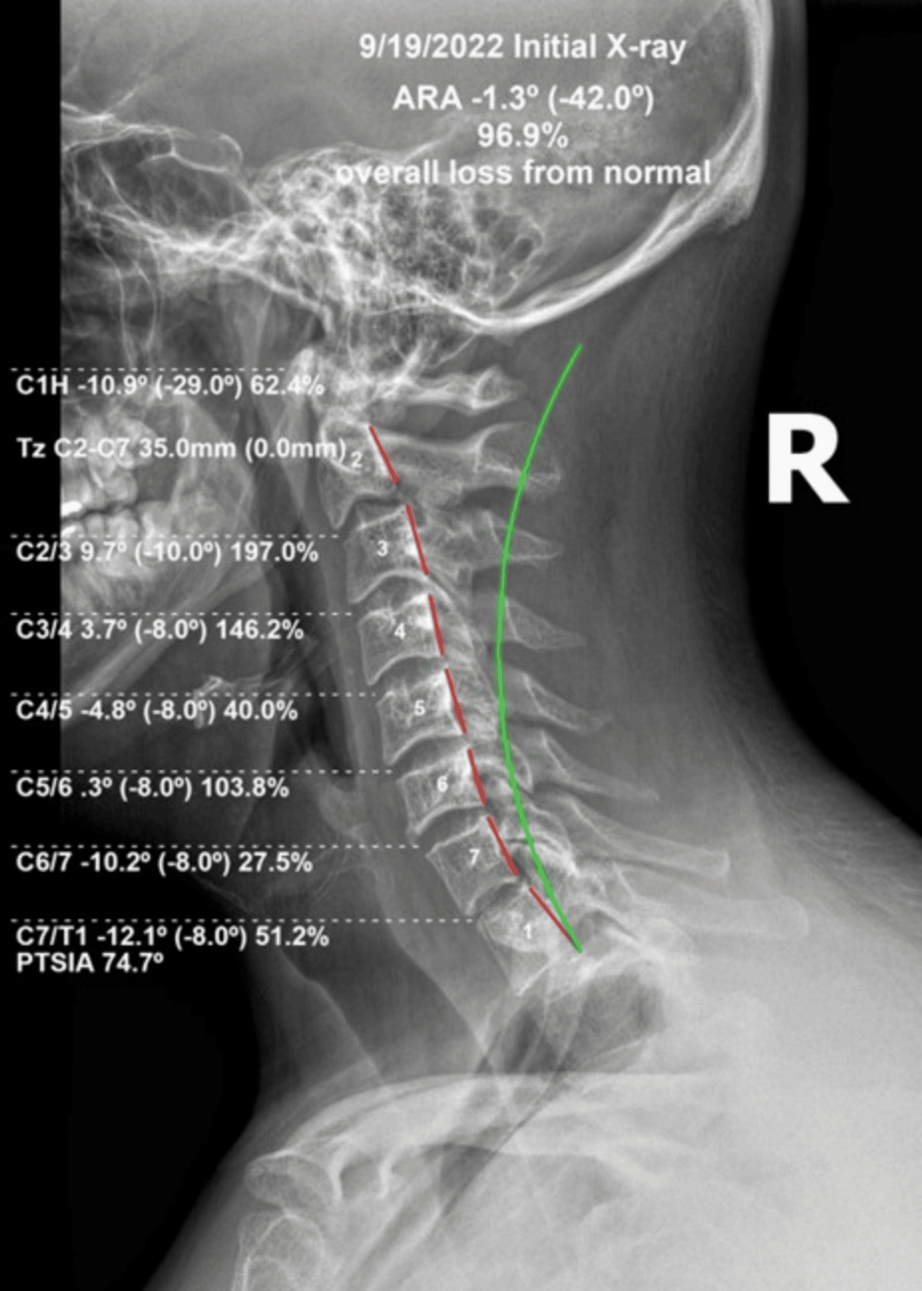
Upright lateral cervical radiograph at the initial examination. The red lines outline the posterior vertebral body margins. The measurements above show the absolute rotation angle (ARA) of -1.3° where a more ideal lordosis would approximate -42° from C2 to C7 (green line). The measurements on the left demonstrate the individual relative rotation of each pair of vertebrae; the patient’s values are listed outside the brackets, and the ideal values are listed inside the brackets.

Standing coronal and sagittal spine radiographs were acquired by the treating physician at a spine facility in Gillette, Wyoming, USA. All state and federal guidelines for radiograph acquisition were applied. The initial radiographs acquired on September 19, 2022, were analyzed digitally and compared with ideal parameter values. Abnormalities in the cervical curvature parameters were noted with a severe kyphotic deformity from C2 to C3 (+9.7° vs. -10° ideal [9]), where the overall lordosis from C2 to C7 was -1.3° compared to normal values between 34° and 42° using the Harrison posterior tangent method (HPTM) [9,10]. A forward flexion abnormality of the head compared to the torso (+RxH) was observed. C1 to horizontal measured a 62.4% flexion abnormality at -10.9° compared to the normal-to-ideal models of upper cervical extension of -24°-29° [9,10]; anterior translation of the head (+TZH) was significant with forward head posture (FHP), measuring 35 mm compared to an upper limit of “normal” at 15 mm [10]. Relative rotation angles at C2-C3, C3-C4, and C5-C6 all showed more than 100% abnormal position, exceeding the tolerance of lordotic spinal alignment and nearly kyphotic deformity overall. Endplate degeneration and osteophytic development were found at the C6-C7 disk level (Figure 1).

Orthopedic assessment and objective outcome measure findings

Pain in the base of the skull was the most common and frequent symptom, and the musculature was very sensitive to any palpation. Central neck and lower back pain was reported by the patient during orthopedic cervical compression testing. Visual ROM to evaluate pain found severe restriction with pain during cervical and lumbar flexion, lumbar extension, and lateral bending of the neck and torso, which also elicited pain in the cervical and lumbar spine and paraspinal muscle. Palpation of the cervical, thoracic, and lumbar spine found taut and tender muscular fibers through all regions with significant tenderness in the cervical and especially upper cervical regions. Sensory pinwheel testing found hypoesthesia at the C5/C6 dermatome. The total quadruple visual analog scale (QVAS) for cervical spine pain measured 53/100 initially, and of note, pain at worst was reported to be 8/10. The headache disability index (HDI) measured 38% [11], and the neck disability index (NDI) [12] measured 38% indicating moderate disability. QVAS for the lumbar spine also showed significant pain levels (53/100), as shown in Tables 1-3. Posture analysis found a left head-to-thorax translation (+T_x_^H^), anterior thoracic translation (+T_z_^T^), and pelvis-to-feet right anterior rotation (+R_y_^P^). Orthopedic cervical compression testing was positive for NP and low back pain.

**Table 1:** Quadruple visual analog scale (QVAS) for the cervical spine: initial, re-assessment, and follow-up scores with overall change

Evaluation	QVAS-cervical				
Date	Current pain	Average pain	Pain at best	Pain at worst	Total
September 19, 2022	4/10	4/10	2/10	8/10	53/100
January 11, 2023	0/10	1/10	1/10	2/10	10/100
July 31, 2023	0/10	0/10	0/10	2/10	7/100
Overall change	4/10	9/10	2/10	6/10	46/100

**Table 2:** Headache disability index (HDI) reporting change of headache on emotional and functional health parameters. Scores of 2-32, 33-59, and greater than 60 represent mild, moderate, and severe disability, respectively.

Evaluation	HDI		
Date	Emotional	Functional	Total
September 19, 2022	12/52	20/48	38/100
January 11, 2023	2/52	6/48	8/100
July 31, 2023	8/52	4/10	12/100
Overall change	4/52	22/48	26/100

**Table 3:** Quadruple visual analog scale (QVAS) for the lumber spine: initial, re-assessment, and follow-up scores with overall change

Evaluation	QVAS-lumbar				
Date	Current pain	Average pain	Pain at best	Pain at worst	Total
September 19, 2022	4/10	4/10	2/10	8/10	53/100
January 11, 2023	0/10	2/10	1/10	3/10	17/100
July 31, 2023	0/10	2/10	0/10	5/10	23/100
Overall change	4/10	2/10	2/10	3/10	30/100

Treatment protocols and frequency

**Figure 2:**
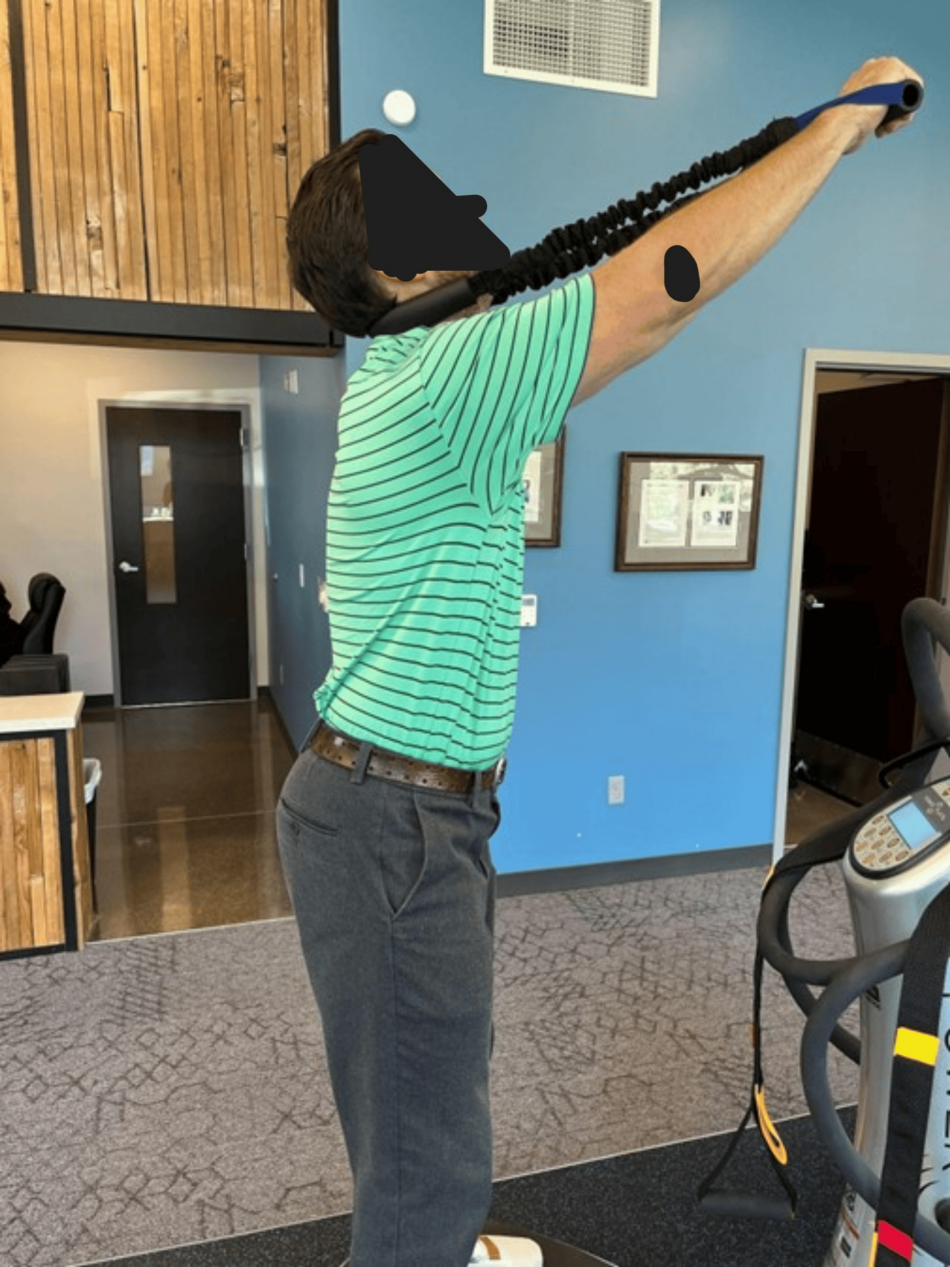
A standing exercise involving cervical extension and head posterior translation with the ProLordotic® resistance trainer under the influence of whole-body vibration with the Power Plate® designed to strengthen the cervical muscular in the mirror image (MI®) of the patient’s initial cervical hypolordosis.

The patient was treated with CBP® structural rehabilitation and orthopedic protocols [13-15], supervised and applied by the evaluating physician over eight weeks for a total of 24 treatments. Mirror image (MI®) cervical extension strengthening exercises were performed using the ProLordotic™ cervical spine exerciser (Circular Traction, Inc., Huntington Beach, CA) applied to the upper cervical spine. These exercises were conducted on a whole-body vibration (WBV) Power Plate® (Power Plate Inc., Northbrook, IL) device [16], as shown in Figure 2.

**Figure 3:**
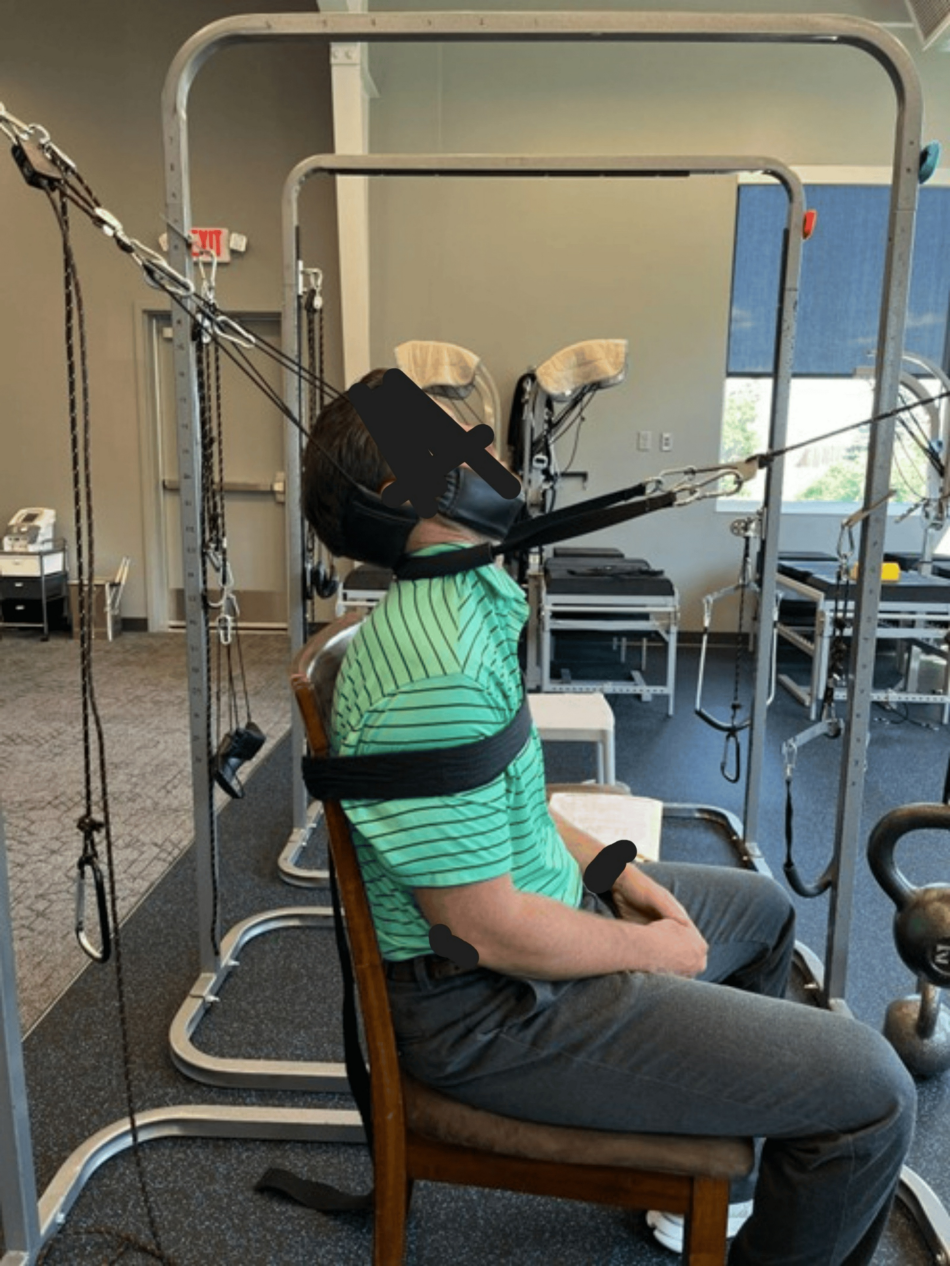
Mirror image® cervical extension traction. The patient is seated, and the torso is restrained with a Velcro strap. Two forces are applied to the cervical spine with the patient’s neck in extension. The posterior pull prevents the patient’s head from being pulled forward, and the anterior pull is applied through the kyphotic deformity.

Additionally, MI® extension traction was applied to target the visco-elastic ligamentous structures around the spine which do not have contractile abilities like muscles and require prolonged application of forces in the correct MI® direction to correct these spinal abnormalities, i.e., to increase the lordosis. Traction began at two to three minutes, and the patient progressed to as much as 15 minutes based on the patient's ability to tolerate the traction (Figure 3). Specific SMT was applied based on the radiographic misalignments and in the direction opposite to the misalignments, using SMT under the posterior neck below the kyphotic deformity while an anterior-to-posterior thrust was performed. According to the radiographs, the SMT was used to increase the ROM and mobility while decreasing pain in the most appropriate direction.

The patient received in-office treatment and was directed to follow home-care procedures, including postural exercise, postural ergonomics education, minimizing pain catastrophizing, and the prescription of a simple device to assist in-office traction (Denneroll™ cervical traction orthotic (DCTO)).The patient was instructed to perform the postural exercises three times per week for 15 seconds per repetition and up to as many tolerable repetitions as possible. DCTO was initiated at two to three minutes and progressed to 15 minutes with placement in the upper neck three times per week. The patient had high compliance with in-office therapy and home-care protocols. Re-evaluation included spine radiography, all health-related outcome measures, and QVAS. No adverse outcomes were reported by the patient, who had high satisfaction with the treatment.

Results

Reevaluation of Subjective and Objective Clinical Findings

Re-evaluation was performed by the treating physician after 24 in-office treatments consisting of MI® exercises, traction, and postural SMT. All orthopedic and neurological testing including cervical compression and sensory pinwheel testing was negative. It was self-reported by the patient that the CM pain/symptoms and prior disability were improved by over 90%. Initial symptoms including muscle weakness, numbness, and loss of function were resolved 100% as reported by the patient. In the rare event that the patient would have a migraine, the aura, pain, and concomitant symptoms were much less severe, and the cramping in the posterior body musculature was resolved. Neck stiffness was reported to be completely resolved, and visual subjective ROM analysis for pain found no abnormalities in all regions. The visual postural analysis demonstrated that only the left thoracic translation (+TxT) remained.

**Figure 4:**
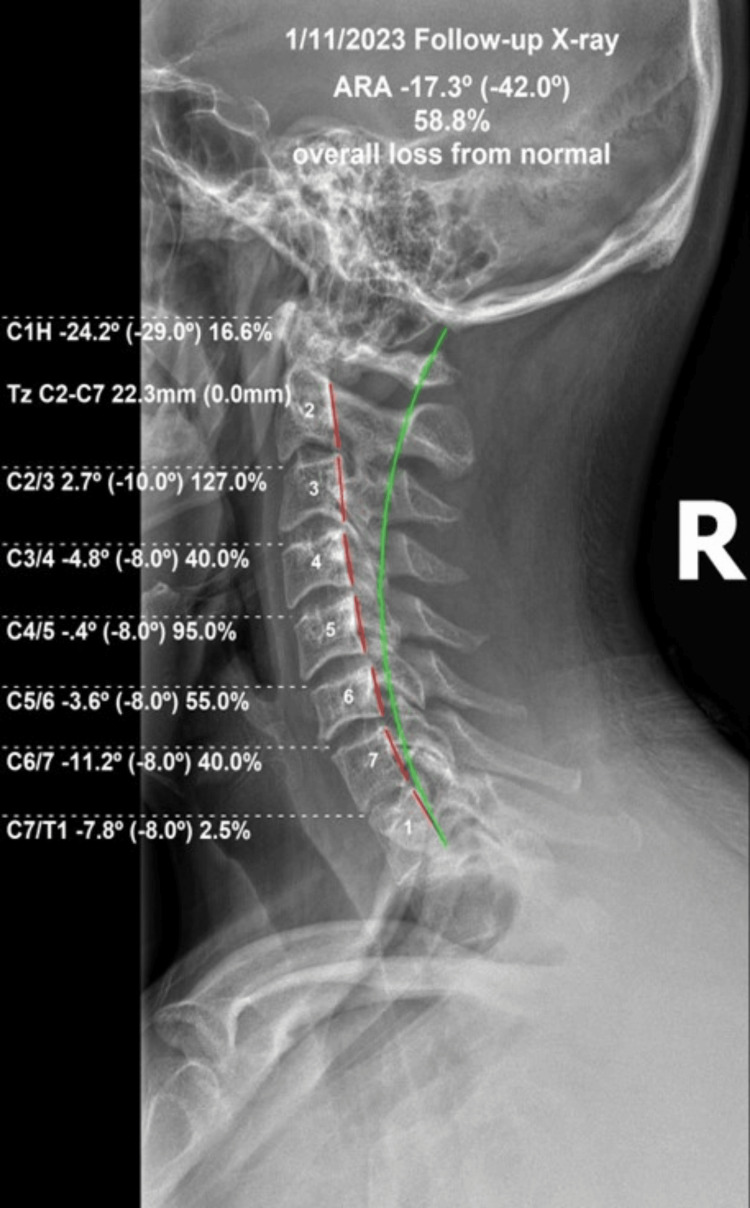
Post-treatment re-examination lateral cervical radiograph following 24 in-office treatments using CBP® protocol. The cervical lordosis improved to -17.3° (vs. -1.3° initially), the atlas plane line improved to -24.2° (vs. -10.9° initially), and the anterior head translation reduced to 22.3 mm (vs. 35 mm initially). The kyphotic C2-C3 segment improved to +2.7° (vs. +9.7° initially). CBP®: Chiropractic BioPhysics®.

All pain and disability scores improved, and any prior medications were no longer used. The cervical QVAS was 10 (vs. 53), the HDI was 8% (vs. 38% initially), the NDI was 4% (vs. 38% initially), and the lumbar QVAS was 17 (vs. 53 initially). Follow-up X-ray examination was performed more than 24 hours after the previous treatment to prevent any residual effects of spine positioning from the prior treatment. The lateral cervical curve C2-C7 improved from -1.3° to -17.3°. The segmental C2-C3 kyphotic relative rotation angle (RRA) improved from +9.7° to +2.7°. The atlas plane line normalized from the initial measurement of -10.9° to -24.2°. The forward head translation was reduced from 35 to 22.3 mm (Figure 4).

Follow-Up Findings

A seven-month follow-up exam was performed after the in-office treatment twice per month and weekly use of the DCTO three times per week at home. The patient continued with home postural and strengthening exercises.This assessment included all baseline physical examinations, and all subjective and objective outcomes were repeated. The patient continued to have no CM symptoms and reported high satisfaction with the in-office and home treatment.

**Figure 5:**
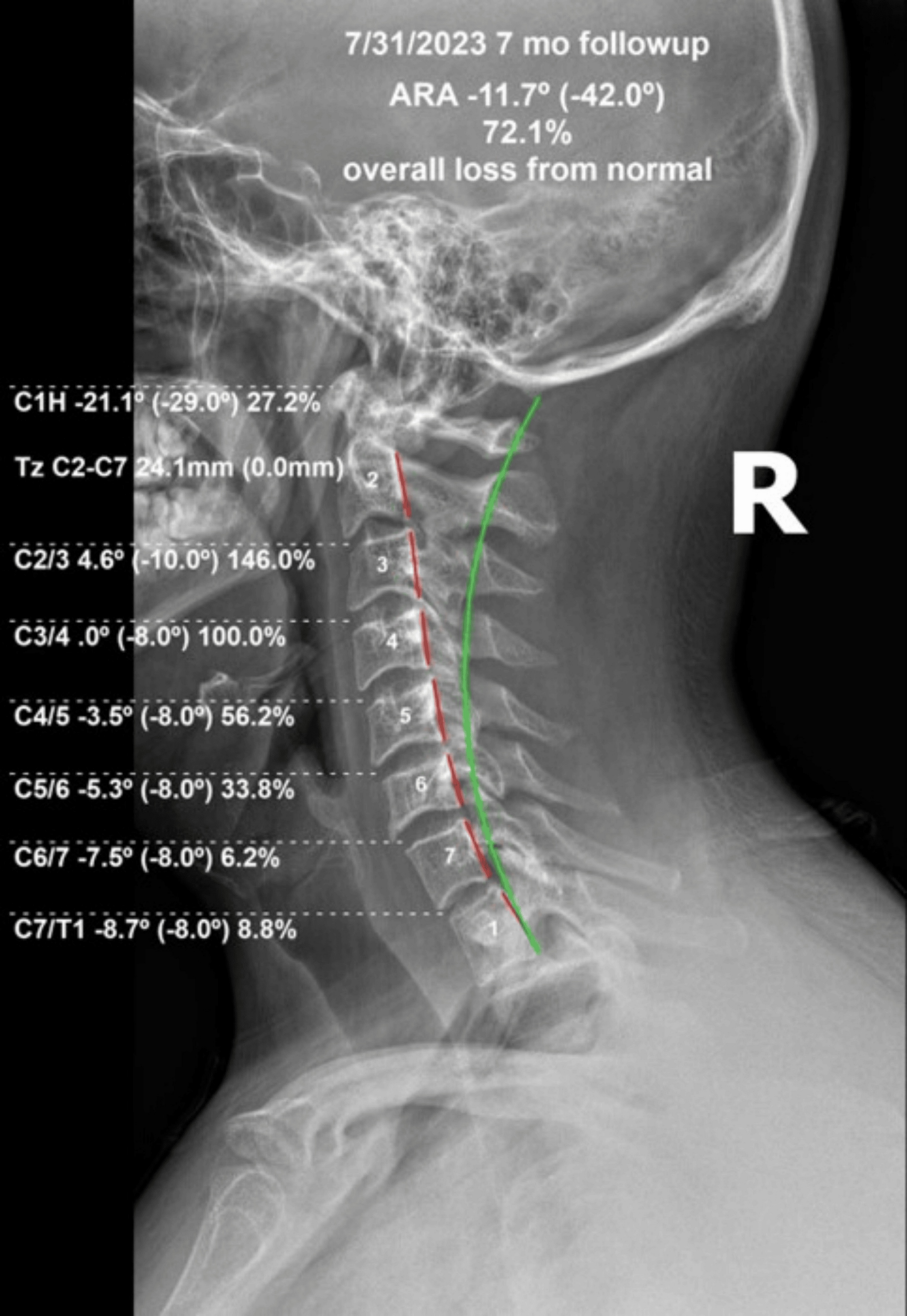
Follow-up re-examination lateral cervical radiograph after seven months of maintenance treatments. The cervical lordosis lost some of the initial correction and was -11.7° (vs. -1.3° initially), the atlas plane line was maintained at -21.1° (vs. -10.9° initially), and the anterior head translation maintained at 24.1 mm (vs. 35 mm initially). The kyphotic C2-C3 segment measured +4.6° (vs. +9.7° initially).

The pain and disability scores showed maintenance of the initial improvements (Tables 1-3). The cervical lordosis lost some of the initial correction and was -11.7° (vs. -1.3° initially), the atlas plane line was maintained at -21.1° (vs. -10.9° initially), and the anterior head translation was maintained at 22.3 mm (vs. 35 mm initially). The kyphotic C2-C3 segment measured +4.6° (vs. +9.7° initially), as shown in Figure 5.

## Discussion

We report a case of a 29-year-old male who had suffered from CM for 19 years and experienced its alleviation with a 16° increase in C2-C7 cervical lordosis. CBP treatment was provided for two months, and the symptom resolution was maintained at a seven-month follow-up, although some initial lordosis correction was lost.

This case report of a patient with CM having clinically significant and measurable improvement in patient-reported outcomes and radiographic spine alignment improvements adds to the few cases that show efficacy for treating headaches using CBP® protocols [7,8]. Loss of the cervical lordosis and altered cervical alignment parameters appear to have played an important part in the patient’s non-improvement in CM with prior treatments [16,17]. However, as lordosis improved with treatment, a simultaneous improvement in CM occurred. Although this case cannot prove causality, the loss of normal cervical lordosis has been suggested as a potential causative factor in the worsening of the duration and intensity of headaches [17].

Prior CM studies in the literature that report positive outcomes resulting from drug therapies are rare [3,4]. Large randomized controlled trials (RCTs) of pharmacologic treatments for use were not found, and studies reporting any long-term conclusions that the drugs are successful as a lifetime treatment without reduction in success or side effects are rare [1-4]. Long-term monitoring of patients with CM is recommended because of the risk of worsening symptoms, such as infarction, seizure, and mental health issues that can have life-threatening consequences.

Conservative non-drug studies in the medical literature documenting successful conservative or pharmacologic treatment of CM with long-term follow-up and high-quality health-related QoL (HRQoL) measures were not found in the literature. However, the reduction of NP and headaches should be a priority for clinicians as this will reduce GBD. Although this is just a case report, it is consistent with two other cases [7,8]. Further, the results from our case are consistent with clinical trials on NP patients [14], so it could be argued that improving the biomechanical alignment of the cervical spine is important for the successful treatment of craniocervical conditions including CM.

Although the exact mechanism for CM is not known, it is known that cervical kyphosis can elicit abnormal stresses and strains onto the soft tissues including the neurological elements. This postural and structural deformation of the central nervous system (CNS), spinal canal, and spinal cord is associated with abnormal production of nociceptive afferentation and inflammatory mediators caused by the breaking of hydrogen bonds under abnormal loads. The consequent cellular and tissue changes result in fibrosis and increase the likelihood of pain and dysfunction [18-20]. The improvement in lordosis, therefore, would result in the removal of strains on the soft tissue elements and then would lead to symptom reduction. This is a possible mechanism of how lordosis improvement may lead to symptom improvements.

Physicians and therapists should seek the most economical and easily applied therapies for simple conditions and complex diseases based on quality case reports, case series, and RCTs demonstrating consistent results. CM is relatively rare, and large RCT studies are unlikely. Therefore, smaller studies like this case will provide physicians with a potential therapeutic approach and promising preliminary results. Further research is indicated to explore these findings more comprehensively.

## Conclusions

This study reports a case of successful non-drug treatment of CM in a 29-year-old male. Migraine affects a significant portion of the population, and the CM subset condition has significant implications for patients including loss of quality of life, decreased work productivity, and a contribution to the GBD. Conservative treatment options are few beyond medication, and this study provides clinicians, physicians, and therapists an option with potentially beneficial effect. Further, larger randomized studies of these methods are necessary to determine the most appropriate treatment for CM. This study adds to the total body of knowledge by demonstrating that the unique condition of CM can be improved subjectively and objectively with a conservative application.
